# Transient Existence of Circulating Mesenchymal Stem Cells in the Deep Veins in Humans Following Long Bone Intramedullary Reaming

**DOI:** 10.3390/jcm9040968

**Published:** 2020-03-31

**Authors:** Sarah M Churchman, Elena A Jones, Tarek Roshdy, George Cox, Sally A Boxall, Dennis McGonagle, Peter V Giannoudis

**Affiliations:** 1 Leeds Institute of Rheumatic and Musculoskeletal Medicine, University of Leeds, Leeds LS9 7TF, UK; s.m.churchman@gmail.com (S.M.C.); e.jones@leeds.ac.uk (E.A.J.); drtarec@yahoo.com (T.R.); georgeldcox@googlemail.com (G.C.); S.a.boxall@leeds.ac.uk (S.A.B.); D.G.McGonagle@leeds.ac.uk (D.M.); 2 NIHR-Leeds Musculoskeletal and Biomedical Research Center, Chapel Allerton Hospital, Leeds Teaching Hospital NHS Trust, Leeds LS7 4SA, UK; 3 Academic Department of Trauma and Orthopaedics, Leeds General Infirmary, Leeds LS1 3EX, UK

**Keywords:** circulation, MSC’s, femoral, peripheral blood, reaming, long bones

## Abstract

The biology of mesenchymal stem cells (MSCs) in humans is incompletely understood and a possible role of systemically circulating cells in health and autoimmune disease remains controversial. Physiological movement of bone marrow MSCs to sites of injury would support the rationale for intravenous administration for relocation to damaged organs. We hypothesized that biophysical skeletal trauma rather than molecular cues may explain reported MSC circulation phenomena. Deep-femoral vein (FV) and matched peripheral vein blood samples (PVBs) were collected from patients undergoing lower-limb orthopaedic procedures during surgery (tibia using conventional sequential reaming, *n* = 9, femur using reamer/irrigator/aspirator (RIA), *n* = 15). PVBs were also taken from early (*n* = 15) and established (*n* = 12) rheumatoid arthritis (RA) patients and healthy donors (*n* = 12). Colony-forming unit-fibroblasts (CFU-Fs) were found in 17/36 FVBs but only 7/74 PVBs (mostly from femoral RIA); highly proliferative clonogenic cells were not generated. Only one colony was found in control/RA samples (*n* = 28). The rare CFU-Fs’ MSC nature was confirmed by phenotypic: CD105^+^/CD73^+^/CD90^+^ and CD19^−^/CD31^−^/CD33^−^/CD34^−^/CD45^−^/CD61^−^, and molecular profiles with 39/80 genes (including osteo-, chondro-, adipo-genic and immaturity markers) similar across multiple MSC tissue controls, but not dermal fibroblasts. Analysis of FVB-MSCs suggested that their likely origin was bone marrow as only two differences were observed between FVB-MSCs and IC-BM-MSCs (*ACVR2A*, *p* = 0.032 and *MSX1*, *p* = 0.003). Stromal cells with the phenotype and molecular profile of MSCs were scarcely found in the circulation, supporting the hypothesis that their very rare presence is likely linked to biophysical micro-damage caused by skeletal trauma (here orthopaedic manipulation) rather than specific molecular cues to a circulatory pool of MSCs capable of repair of remote organs or tissues. These findings support the use of organ resident cells or MSCs placed in situ to repair tissues rather than systemic administration.

## 1. Introduction

Bone marrow (BM) mesenchymal stem cells (MSCs) are progenitors with multipotential capacity for tissue repair and are defined by plastic adherence with colony-forming nature, the ability to form cartilage, fat and bone, and have a defined immunophenotype [1]. MSCs are located in many skeletal tissues such as bone [2], BM [3], synovial fluid [4], synovium [5], fat pad [6], cartilage [7] and dental pulp [8] as well as in other locations around the body such as adipose tissue [9], tendon [10], umbilical cord [11] and early trimester umbilical cord blood [12].

In humans there is evidence of MSC circulation in early foetal development [13] but their circulation in later development and life remains contentious [14,15,16].

Several animal studies have suggested that MSCs circulate either naturally (mouse/rabbit/guinea pig) [17], under hypoxia (rat) [18] or electro-acupuncture (rat) [19], or through growth factor (mouse [20]/human [21]) stimulation. One study demonstrated that “connective tissue progenitors” homed to fractured femurs in the parabiotic mouse [22], whereas another parabiotic study failed to show evidence of circulation [23]. Nonetheless, a recent review supported systemic migration to sites of inflammation in rodents [24]. Human studies have shown no evidence for MSCs circulating in the periphery of patients with organ injury [25], although MSCs were reported following severe trauma and skin damage [25,26,27,28], but not always [29].

Culture-expanded MSCs have been injected into venous circulation in the belief that MSCs home to sites of injury or inflammation and thus participate in tissue repair, however, with the exception of an immunomodulatory effect, evidence that MSCs fulfil this role is not strongly supported. A pathogenic role for circulating MSCs has also been postulated for autoimmune diseases such as rheumatoid arthritis (RA), a polyarticular condition where abnormal synovial fibroblasts contribute to joint destruction [30,31,32]. Putative MSCs were identified in human RA joint synovial tissues [33], and reportedly infiltrated joint cavities early in a collagen induced arthritis murine model via BM vascular canals, although this was not functionally demonstrated [31]. The same group reported putative mesenchymal precursor cells in blood, although strict phenotypic criteria were not applied, and suggested their link to RA [32]. Indeed, a xenogeneic mouse model suggested that RA synovial fibroblasts can “migrate” large distances following subcutaneous injection, presumably via the circulation, and contribute to pannus-mediated cartilage destruction [30].

Methodological limitations of previous studies generally relate to a lack of strict functional, phenotype and molecular criteria for what defines an MSC. In healthy individuals previous studies found “fibroblast-like” cells which were not MSCs [14,34] and other studies have claimed “MSC-like cells” although these did not exhibit characteristic MSC proliferation or plastic adherence [32,35].

Given that fat embolization may occur after skeletal trauma [36] and that MSCs associate with adipocytes [37], we hypothesized that MSCs might circulate as a direct consequence of skeletal injury rather than molecular cues. Accordingly, MSCs might be more abundant in the deep veins following certain orthopaedic procedures or trauma. The present study used strict phenotypic and molecular criteria for MSCs and investigated: 1) whether MSCs could be found in the adult human circulatory system in health or RA (representing autoimmune disease), 2) whether MSCs circulate following trauma (induced by intramedullary reaming) and can reach the peripheral circulatory areas, and 3) the likely origin of such circulating cells.

## 2. Materials and Methods

### 2.1. Donors and Tissue Collection

All tissues were donated following informed written consent with ethical approval (06/Q1206/127 and 04/Q1206/107) from the National Research Ethics Committee (Yorkshire and Humberside). Blood samples (Table 1) were taken from patients undergoing planned orthopaedic surgery or attending routine outpatient clinics (patient information in Supplementary Table S1).

Prior to initiation of surgery, patient positioning (supine) was adjusted to facilitate easy access of the femoral vein puncture during the procedure. Under sterile conditions, 10ml of femoral venous blood from the limb ipsilateral to the surgical procedure was obtained by routine venepuncture straight after the reaming with reamer/irrigator/aspirator (RIA) [38]; in contrast, in patients where sequential reaming was performed using standard fluted reamers, samples were obtained after the second reaming step (Depuy Synthes, Paoli, PA, USA). Peripheral (antecubital) vein blood (PVB) samples were taken immediately afterwards in both groups. Femoral vein blood (FVB) was aspirated using a 20ml syringe with a 12 gauge needle and immediately transferred to EDTA vacutainers to prevent clotting. PVBs were collected from patients with early RA (<1year duration) and established RA; patients met the EULAR/ACR criteria (Supplementary Table S2). Further PVB collections were made from healthy control (HC) volunteers. Femoral blood could not be taken from HC and RA groups due to ethical constraints.

MSCs from “local” control tissues (tissues penetrated by the introduction of a reamer) for the molecular study were long-bone-BM directly harvested from the bone canal by inserting a feeding tube for aspiration prior to reaming (*n* = 10, 4 females), healthy bone (pelvis) (*n* = 11, 5 females) and periosteum (*n* = 7, 2 females). Passage 1 adipose tissue MSCs purchased from Life Technologies (*n* = 3, 2033, 2117, 2118), Zenbio (*n* = 2, ASC0046, ASC0049) and Lonza (*n* = 1, 407088) (5 females, median age 47.5, range 29–63) and negative control skin fibroblast cell lines (*n* = 6) to exclude skin or perivascular tissue. Iliac crest (IC)-BM aspirated from healthy/trauma patients (*n* = 10, 5 females), was used as a commonly studied gold-standard MSC comparator.

### 2.2. Preparation of Tissue and Generation of MSC Cultures

Since plating of whole blood can lead to clot formation, mononuclear cells (MNCs) were isolated from whole blood and BM using Lymphoprep (Axis-Shield, Dundee, UK) density gradient centrifugation, accepting that some MSCs could theoretically be missed. All MNCs were plated; a 2 × 10^6^/10 cm dish was used for colony-forming unit-fibroblast (CFU-F) assays or expansion. Bone and periosteum were digested using 600 U/mL collagenase (Worthington Biochemicals, Lakewood, US), 20% fetal calf serum (PAA laboratories, Little Chalfont, UK) in phosphate-buffered saline and incubated at 37 °C for 4 h with intermittent agitation; 1 × 10^5^ cells were seeded per dish. Bone, IC-BM, long-bone-BM and blood cells were all expanded for 3–4 weeks in 15 ml NH medium (Miltenyi Biotec, Bisley, UK) with twice weekly half media changes. Adipose-MSCs and fibroblasts were revived and expanded to p2 in their manufacturers’ recommended media.

### 2.3. Colony-Forming Unit-Fibroblast Assay

After 14 days, CFU-F dishes for the FVBs and PVBs were terminated and the dishes stained using crystal violet [39]. Colonies (>50 cells), when present, were normalised to the volume of blood collected or the number of MNCs seeded. For non-terminated dishes, visual inspection was undertaken, and any colonies recorded.

### 2.4. Phenotypic Analysis of Putative Blood-Derived MSCs

Colonies expanded from blood were trypsinised, washed and stained with antibodies: CD31-FITC, CD90-PE, CD105-PE (AbD Serotec, Kidlington, UK), CD19-PE, CD33-FITC, CD34-PerCp, CD45-PE-Cy7, CD61-FITC, CD73-PE, (BD Biosciences, Oxford, UK) and CD271-APC (Miltenyi Biotec), at manufacturers recommended concentrations. Cells were washed, 30,000 events captured on a LSRII flow cytometer and the data analysed using FACSDiva Software (both BD Biosciences).

### 2.5. Osteogenic Differentiation Assay

Differentiation was performed by seeding 3 × 10^4^ cells into 3 cm tissue culture dishes. Following two weeks in osteogenic medium (with twice weekly changes), alkaline phosphatase activity was visualised [39].

### 2.6. Gene Expression of MSCs

Culture-expanded MSCs from all tissue types were washed and lysed directly in their dishes. RNA was extracted using RNA/DNA/protein kit (Norgen Biotek, Thorold, Canada) and cDNA transcribed with High Capacity Reverse Transcription kit (ThermoFisher, Warrington, UK). Taqman assays (Supplementary Table S3) were run according to manufacturer’s recommendations in Format 96a Taqman low density arrays on a 7900HT system (ThermoFisher). Expression is reported relative to *HPRT.*

### 2.7. Hierarchical Clustering

An open-source cluster 3.0 [40] was used for hierarchical clustering of gene expression data. The filters used were 80% presence of gene expression, log transformation, genes and arrays which were clustered with their weights calculated using uncentered correlation. Complete linkage clustering was then performed. Output was viewed/displayed in Java TreeView [41].

### 2.8. Statistical Analysis

Presence or absence of CFU-Fs was tested using a Chi-squared test. Comparison of gene expression from different MSC sources was tested using Kruskal–Wallis followed by Dunn-Bonferroni post-hoc multivariate test when *p* < 0.05. Long-bone-BM-MSC and IC-BM-MSC similarity was tested using Mann–Whitney U (IBM SPSS statistics 21).

## 3. Results

### 3.1. Identification and Frequency of MSC-Like Colonies

**Figure 1:**
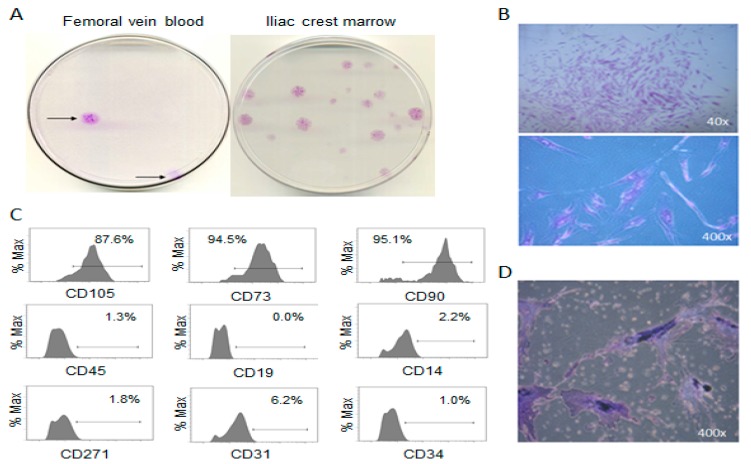
Identification and phenotype of putative MSC colonies. (**A**) Macroscopic image of fibroblast-like colonies isolated from femoral vein blood (FVB) compared to iliac crest bone marrow, (**B**) microscopic images of FVB colonies, (**C**) histograms from flow cytometry of plastic adherent culture-expanded cells isolated from FVB (**D**) alkaline phosphatase staining of putative MSCs following osteogenic induction.

Primarily, FVB and PVB of patients undergoing long-bone surgical reaming yielded plastic adherent CFU-Fs with the same appearance as the positive control IC-BM-MSCs (Figure 1A,B). These putative MSCs possessed the accepted [1] surface phenotype (Figure 1C) and proliferated adequately for osteogenic differentiation demonstration (Figure 1D), but not chondro- and adipo- genesis assays.

### 3.2. Circulatory CFU-Fs in Femoral and Peripheral Veins.

**Figure 2:**
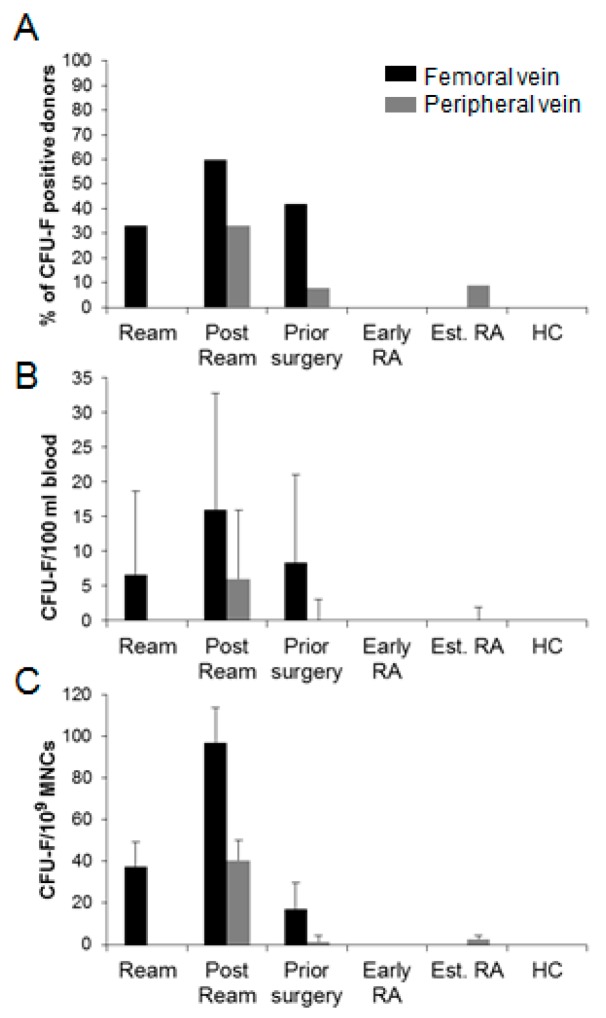
Frequency of MSC from different donor cohorts. Cohort specific: (**A**) percentage of donors yielding at least one CFU-F; (**B**) CFU-F/100 ml blood; (**C**) CFU-F/10^9^ MNCs. Error bars are standard deviations.

Overall, CFU-Fs occurred in 17/36 FVBs and 7/74 (matched for surgical patients) PVBs; a total of 47 colonies from 1.716 litres of blood. Colonies were present in 12/24 FVBs of patients undergoing reaming, however, reaming of the femur (RIA) yielded colonies in sixty percent of donors as opposed to thirty-three percent when conventionally reamed (not significant; Figure 2A). Additionally, greater CFU-F/100 ml blood (and CFU-F/10^9^ MNCs) in the reamed femur FVBs compared to other surgical groups (Figure 2B) suggested greater liberation of MSCs from the femur, but that change was inconsequential in relation to the MNC number (Figure 2C). Evidence did suggest that CFU-Fs may persist in the event of unstable fracture or metal fixation, at least in the local area (present in 5/12 patients requiring further surgery).

MSCs were absent from PVB following tibial reaming, but present immediately after femoral/RIA reaming (5/15; Chi square *p* = 0.052), but the frequency remained incredibly low (4.0 × 10^–8^ MNC). One colony was isolated from PVB of the prior surgery and established RA groups (1.4 × 10^−9^ and 2.6 × 10^−9^/MNC respectively), but not early RA and HC.

### 3.3. Molecular Profile of Blood-Derived CFU-Fs.

Extensive qPCR analysis was used to measure gene transcripts known to be expressed in culture-expanded MSCs and associated with mesenchymal lineages (in lieu of differentiation), multipotency and other known character traits [39].

MSCs from “local” control tissues, bone, adipose tissue, long-bone-BM and periosteum as well as frequently studied IC-BM and negative control skin fibroblasts, were investigated alongside the blood-MSCs (two or three colonies for each donor).

For statistical analysis, transcripts with enough detection across all MSC types were considered (80 genes, qPCR data not shown in Figures are located in Supplementary Figure S1).

**Figure 3:**
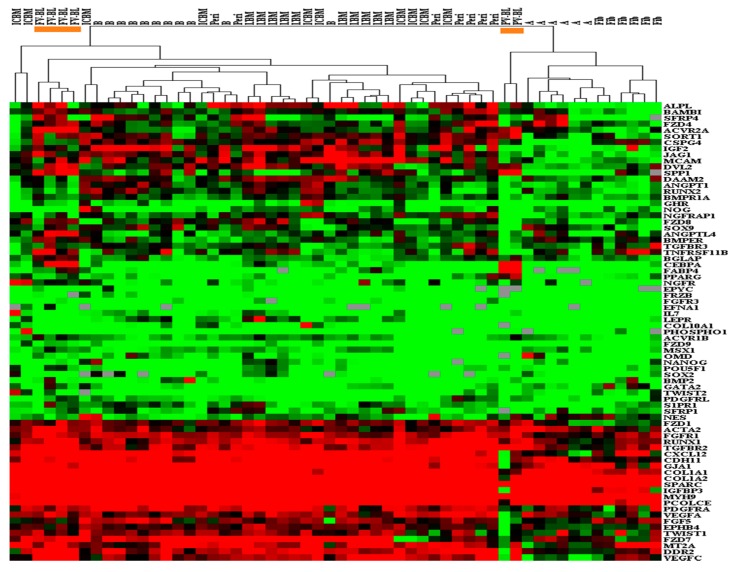
Hierarchical clustering of blood-MSCs and control MSCs. Orange identifies the two areas of blood-MSCs; FV-BL MSCs (left) within the “bone associated” MSC branch, and PV-BL MSCs (right) within the fibroblast and adipose-MSC branch. BM = bone marrow, BL = blood, IC = iliac crest, LBM = long bone marrow, B = control bone, Peri = periosteum, A = adipose.

Primarily, unsupervised hierarchical clustering of data (Figure 3) showed two distinct groupings. One contained skeletal MSCs (IC-BM, long-bone-BM, periosteum and bone) where the FVB-MSCs resided, whilst the other contained all adipose-MSCs and fibroblasts, with PVB-MSCs more related to these, but on an independent branch within this group and towards the skeletal MSCs.

**Figure 4:**
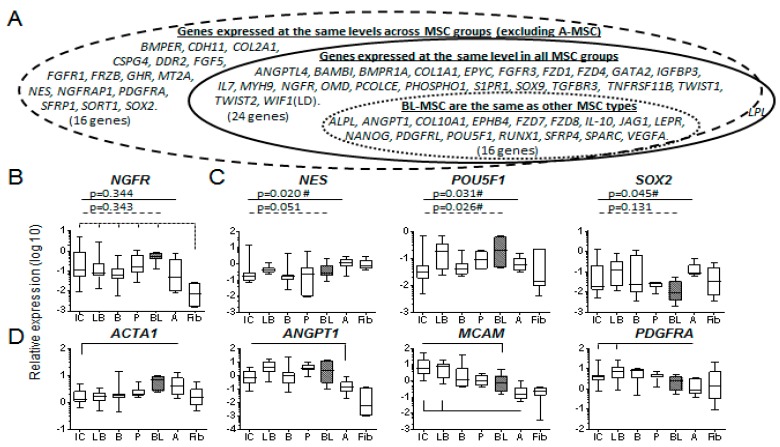
Evidence of molecular similarity of BL-MSCs to other MSC types. (**A**) Representation of gene expression similarity between BL-MSCs and other MSC types. Solid line: same across all MSC groups for 24 genes, dashed line: same for an additional 17 genes (but no longer LPL) when adipose-MSCs were excluded, dotted line: post-hoc multivariate analysis showed that gene expression was the same between blood-MSCs and all other MSC types for a further 14 genes. (**B**) NGFR transcript expression was the same for all MSC types, dashed lines show all MSCs (except adipose-MSCs) distinct from fibroblasts. (**C**) blood-MSCs expressed markers of immaturity at similar levels (Kruskal–Wallis) to other MSC types, no multivariate differences were found between blood and other MSC types. (**D**) Pericyte-related markers were expressed at similar levels across MSC types, any multivariate differences are shown (solid line). IC = iliac crest marrow, LB = long bone marrow, B = control bone, P = periosteum, BL = blood, A = adipose, Fib = fibroblast.

Statistical analysis tested whether relative gene expression levels were the same across all MSC types (not fibroblasts), summarised in Figure 4A, this supported similarity for 24/80 genes tested. Further assessment excluded adipose-MSCs since they clustered with fibroblasts and are considered functionally different from skeletal MSCs [42]. This saw 39/80 genes statistically similar across the remaining MSC groups (*LPL* was no longer the same). Specifically considering the relationship between blood-MSCs and other MSC types, multivariate comparisons deduced an additional 16 genes whose expression was not different in blood-MSCs compared to the other MSC types.

The blood-MSCs expressed *NGFR* (CD271 selection marker) at similar levels to all other MSCs (*p* = 0.343, Figure 4B) and similar levels of nestin/*NES*, *SOX2* and Oct4/*POU5F1*; markers of MSC immaturity (Figure 4C). In terms of pericyte markers, with which IC-BM-MSCs have an overlapping phenotype [43] (Figure 4D) the IC-BM was different from blood with respect to *ACTA1* and *MCAM*, but all were similar for *ANGPT1* and *PDGFRA*. MSCs’ likeness in their chondrogenic potentials was represented by *COL2A1*, *COL10A1*, *EPYC* and *SOX9* as were some markers of osteogenesis: *BMPR1A*, *CDH11*, *OMD*, *SPARC*, *TNFRSF11B* (Supplementary Figure S1). The only adipo-related molecule that showed similarity was *LPL* (with adipose MSCs included) although others showed overlapping expression levels.

Collectively, the origin of the blood-MSCs was difficult to deduce (Table 2, top). Analysis of FVB-MSCs alone suggested that their likely origin was marrow as only two differences each were observed between FVB-MSCs and IC-BM- or long-bone-BM-MSCs (Table 2, all).

**Figure 5:**
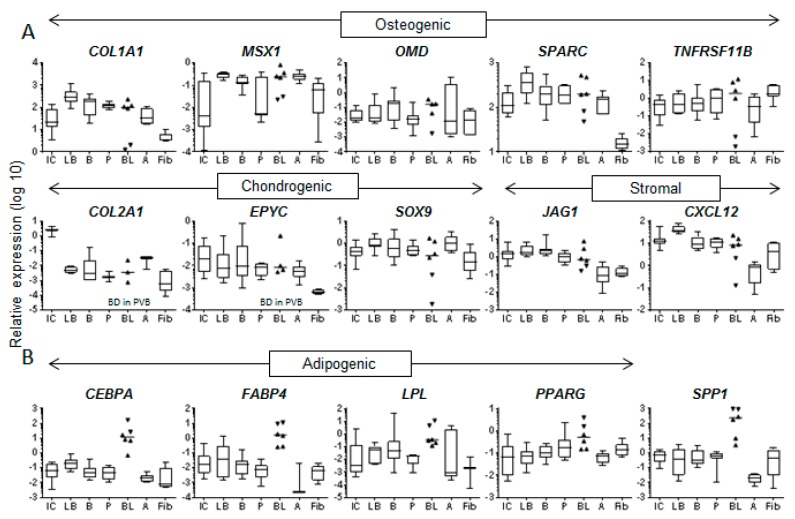
Differential expression of FV- and PV- blood-MSCs compared to other MSC types. **A** – Osteogenic, chondrogenic and stromal-support genes, **B** – adipogenic genes. FVB- and PVB-MSCs represented by standard and inverted triangles, respectively. Horizontal line = median (scatter and box/whisker), box/whisker = interquartile range/range, BD = below detection. IC = iliac crest marrow, LB = long bone marrow, B = control bone, P = periosteum, B = blood, A = adipose, Fib = fibroblast.

Despite statistical similarity, it was common to see a large spread in gene expression for the blood-MSCs (Figure 4 and Figure 5 and Supplementary S1). Closer inspection revealed that for 31 transcripts there was separation between PVB-MSCs and FVB-MSCs (Supplementary Figure S2). When divided into functional categories the osteogenic, chondrogenic and stromal support molecules (Figure 5A) saw PVB-MSCs display lower expression than their FV counterparts.

However, adipogenic markers *CEBPA*, *FABP4*, *LPL* and *PPARG* (together with *SPP1* known to increase in the presence of fat [44] showed higher expression in PVB-MSCs (Figure 5B), possibly pointing towards a close association with fat droplets [37]. The extreme scarcity of these cells did not permit statistical support for PV-specific traits, but the pattern of findings was striking.

## 4. Discussion

The circulation of MSCs in humans is controversial. This study employed a translational model of scheduled orthopaedic skeletal manipulation to test whether trauma might lead to MSC circulation. Phenotypic, limited functional and molecular criteria demonstrated very rare central and peripheral blood MSCs and their likely origin from sites of skeletal damage. These conclusions were based on the occurrence of at least one CFU-F in thirty-three percent of the RIA donors’ PVBs, in addition to twenty-seven percent greater frequency in their FVBs compared to conventional reaming donors (no FVBs were collected before intervention). CFU-Fs were absent in control PVBs (HCs, early and established RA) with the exception of one colony (same as an earlier study [27]), most likely following minor skeletal trauma with occasional MSC escape and circulation.

From their phenotypic and molecular profiles, the circulatory cells described herein are compatible with mesenchymal stem cells, but the extreme rarity of these cells made the formal demonstration of chondrogenesis and adipogenesis impossible. Whilst MSCs and stromal cells are part of the primitive mesenchyme and are essential for the development of the circulatory system [45], these findings challenge the idea that MSCs circulate under normal physiological conditions suggesting that MSC biology is independent of the circulatory system, unlike haematopoietic SCs that are known to circulate, and can be numerically increased for therapeutic purposes. In murine models of rheumatoid arthritis, a destructive polyarthritis, migratory stromal cells reportedly carry the disease to distant sites, presumably via the circulation [30]. Here, given the number of cases and the total volume of RA blood examined, the presence of a single colony in an established RA patient makes it difficult to link these cells into pathogenic models for autoimmune diseases.

Previous studies in orthopaedic settings showed mixed results. CFU-Fs were found in hip fracture patients pre- and post-surgery, but not 6–8 weeks later [26]. Other studies 1 and 8–9 days post multiple-fractures, reported MSCs [25,27]. A further study “within 24 hours” and up to one year follow-up in poly-trauma (low PBMC seeding) showed no MSCs [29]. In contrast, a more recent study reported that polytrauma patients had partly reduced relative numbers of MSC- like cells in peripheral blood in the time course after injury [46].Nonetheless, this study is limited by the small number of patients recruited (11 patients) and not using colony assays for MSC quantification or their molecular profile [46].

Increased MSC frequencies in patients with severe burns [28] is probable, but the reported normal frequency (0.0078%) [28], casts numerical doubt on MSC exclusivity and therefore physiological function. Claims of MSC presence in 100% of samples at frequencies of 0.3%–0.7% [32], 1%–3% [35] and 2/10 physiologically normal controls [17], is several orders of magnitude higher than anything evident in the present work. Previously utilised phenotyping strategies did not confirm these cells exclusively as MSCs, endothelial cells being another possibility. Considering our findings, the previous results likely depend on the degree of trauma and timing of sample collection that in some settings permits MSC detection following injury.

Molecular profiles of blood-MSCs compared to MSC tissue controls from possible sites of origin were scrutinised, especially to determine whether the CFU-Fs merely represented “irrelevant” tissue fibroblasts trapped in the needle during collection. Across a range of MSC-associated genes [39], the majority were expressed at similar levels, supporting skeletal MSC origin rather than translocated fibroblasts. However, the differential genes provided evidence for marrow being the origin of the FV-MSCs. IC-BM-MSCs also shared similarity, but as an un-disturbed area then, it was an unlikely source and the relationship came from its own similarity to long-bone-BM (*p* > 0.05 for 63/80 genes, data not shown). Gene expression studies pointed to a fatty environment although transcript differences of total blood-MSCs or FV- and PV- MSCs separately demonstrated that they were not the same as adipose-MSCs. It has been previously shown that MSCs do tend towards a more adipogenic phenotype (increased *CEBPA/B/D*, *PPARG*, *FABP4*, *LPL*, *PNPLA2* (ATGL) and *SPP1*) in a fattier environment in both humans and mice [44.47}, and that in keeping with lower osteogenic gene expression exhibit decline in their osteogenicity [47]. Since MSCs are closely associated with fat globules [37,48] it is plausible that these cells are indeed MSCs induced into the pre-adipocyte phase by their environment [48] and that globular fat provides a vector for movement of “fattier” MSCs into the periphery [49].

The findings are not suggestive of the different devices being responsible for the presence of MSCs in the periphery, since the RIA causes negative pressure compared to conventional reaming [50] and therefore the higher pressure of the conventional reamer would more likely cause displacement of cells [51]. However, MSCs with a fatty phenotype being able to transiently enter the peripheral bloodstream can be explained by the following arguments: a) an isolated femoral fracture is more likely to cause fat embolism than a tibial fracture [52]; b) the greater supracondylar venous network of the femur provides more escape routes compared to tibia; c) femur being a more potent haemopoietic site compared to tibia [53] provides higher amount of fat marrow content (more micro-vectors).

The limitation of this study was the extremely small number of cells studied intrinsic to the very low frequency of blood MSCs. Complete trilineage differentiation was not therefore possible, but comprehensive lineage specific transcript analysis was undertaken in lieu. Additionally, although we have recruited a total of 74 participants, our study is limited by the size of the samples stratified in different small groups, which made it difficult to find statistical differences. Nevertheless, such stratification was valuable in order to gain important insights on the tissue origins of the circulating cells.

**Figure 6:**
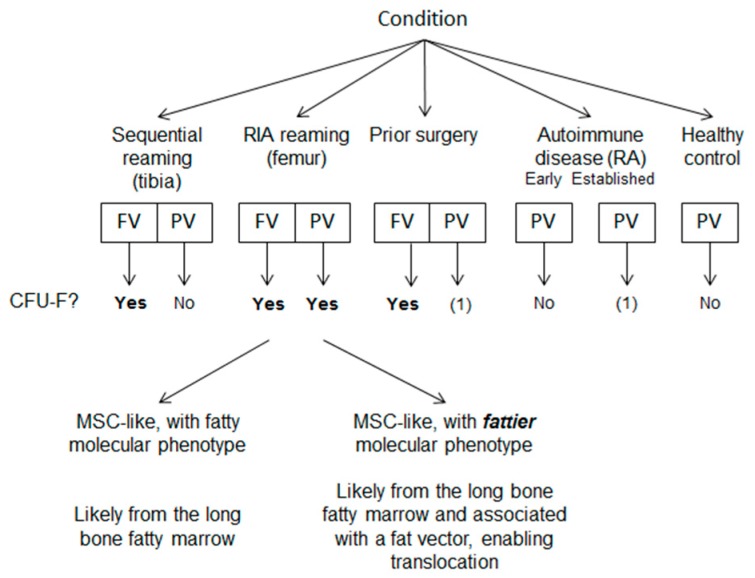
Study-wide summary of CFU-F findings. Yes = CFU-Fs found in multiple donors, No = CFU-F never found in designated group, (1) = only one colony found from all donors in designated group.

To conclude, the present work (summarised in Figure 6) suggests that stromal cells with the phenotypic and molecular characteristics of MSCs can circulate following biophysical skeletal trauma/marrow cavity disruption.

Variation in mechanical factors may explain some discrepancies in the literature. Our findings do not support a role for systemic MSC circulation in RA or other autoimmune diseases and argue against treatment strategies for organ damage based on systemic MSC infusion. Where difficulties in bone repair are anticipated, local implantation of MSC to optimise osteogenesis is desirable. This strategy can be beneficial in the treatment of recalcitrant fracture non-unions and bone defects.

## Figures and Tables

**Table 1:** Blood collection information and total blood volumes used for mesenchymal stem cells (MSC) examination.

Group	Surgery/Sampling Time	Bloods	Total Blood Volumes (mL)	*n*	F:M	Median Age (Range)
Reaming	Patients undergoing reaming	FV+PV	510 + 510	24	10:14	44.5 (18–66)
	a) Sequential conventional reaming for IM nailing of closed tibial shaft fracture.	FV+PV	360 + 360	9	3:6	40 (18–53)
	b) All fracture non-union patients requiring RIA for bone graft acquisition.	FV+PV	150 + 150	15	7:8	45 (19–66)
Prior surgery	Current surgery not relevant ^#^, samples taken before surgical intervention	FV+PV	120 + 120	12	5:7	40 (18–67)
Early RA	N/A	PV	168	15	11:4	51 (23–77)
Established RA	N/A	PV	168	11	7:4	47 (32–66)
HC	N/A	PV	120	12	4:8	33.5 (24–54)
OVERALL		FV + PV	630 + 1086	74	37:37	43.5 (18–77)

F = female, M = male, FV = femoral vein, PV = peripheral vein, RA = rheumatoid arthritis, HC = healthy control, IM = intramedullary, RIA = reamer/irrigator/aspirator. ^#^ available in Supplementary Table S1.

**Table 2:** Differences between blood-MSCs and FVB-MSCs, and other MSC types.

	ICBM-MSCs		LBM-MSCs		CB-MSCs		Peri-MSCs		A-MSCs	
Comparator	BL-MSCs	FVB- MSCs	BL-MSCs	FVB- MSCs	BL-MSCs	FVB- MSCs	BL-MSCs	FVB- MSCs	BL-MSCs	FVB-MSCs
*ACTA2*	0.028									
*ACVR1B*							0.005	0.009		
*ACVR2A*	0.010	0.032			<0.001	0.002				
*BGLAP*			0.006	0.008	0.010	0.014				
*BMP2*									0.012	0.042
*CEBPA*	0.027				<0.001	0.002	0.018		0.001	0.004
*COL1A2*			0.021							
*CXCL12*			0.013							
*DAAM2*			0.016							
*DVL2*	0.041								0.003	0.011
*EFNA1*	0.040									
*FABP4*					0.029		0.008	0.038	0.008	0.020
*FZD9*							0.038	0.008		
*GJA1*			0.037		0.042					
*IGF2*					0.048					
*MCAM*	0.038									
*MSX1*	0.003	0.030					0.050			
*NOG*			0.014							
*PPARG*	0.043		0.041							
*RUNX2*					0.017					
*SORT1*									0.006	
*SP7*									0.008	
*SPP1*			0.017		0.041				<0.001	0.038
*TGFBR3*			0.015		<0.001				0.001	
Differences (BL-MSCs)	8		9		9		5		8	
*ANGPTL4*						0.012				
*BMPER*				0.021				0.031		
*DDR2*						0.046				<0.001
*FGFR1*										0.005
*GHR*										0.012
*PCOLCE*						0.027				0.005
*S1PR1*										0.033
*TGFBR2*						0.002				0.004
*VEGFC*						0.012				<0.000
Differences (FVB-MSCs)		2		2		8		4		12

Top 24 genes are different (*p* < 0.05) across all MSC types (Kruskal–Wallis) when all blood-MSCs (*n* = 6) are considered, the bottom 9 genes are only different (*p* > 0.05) when FVB-MSCs (*n* = 4) are considered independently. Multivariate *p* values < 0.05 are shown. BL = blood, IC = iliac crest, LB = long-bone, CB = control bone, peri = periosteum, A = adipose.

## Supplementary Materials

The following are available online at https://www.mdpi.com/2077-0383/9/4/968/s1, Supplementary Figure S1: 1A (*ACTA1-GATA2*) Relative gene expression in MSCs of different origin and 1B (*GHR-WIF1*) Relative gene expression in MSCs of different origin, Supplementary Figure S2: Thirty-one genes with no overlapping gene expression between PVB- and FVB-MSCs, Supplementary Table S1: Surgical patient information, Supplementary Table S2: Rheumatoid arthritis patient information and Supplementary Table S3: Taqman assays used.
