# Supplementary material for: Transient Existence of Circulating Mesenchymal Stem Cells in the Deep Veins in Humans Following Long Bone Intramedullary Reaming
Source: J Clin Med. 2020 Mar 31;9(4):968. doi: 10.3390/jcm9040968 (PMC7230570; doi:10.3390/jcm9040968)
Supplement: Supplementary file 1 [file jcm-09-00968-s001.pdf]

Supplementary information

Full Title: Transient existence of circulating multipotential stromal cells in the Deep Veins in Man following long bone intramedullary reaming.

Authors: Sarah M Churchman PhD[1,2] , Elena A Jones PhD[1,2],Tarek Roshdy MD[1], George Cox MD[1], Sally A Boxall PhD[1], Dennis McGonagle FRCPI PhD[1,2*#] Peter V Giannoudis MB MD FACS FRCS[2,3#]

CONTENTS

Supplementary Table 1. Surgical patient information

Supplementary Table 2. Rheumatoid arthritis patient information

Supplementary Table 3. Taqman assays used

Supplementary Figure 1A (*ACTA1-GATA2*) Relative gene expression in MSCs of different origin

Supplementary Figure 1B (*GHR-WIF1*) Relative gene expression in MSCs of different origin

Supplementary Figure 2 Genes with no overlapping gene expression between PVB- and FVB-MSCs

Supplementary Table 1. Surgical patient information

Surgical Group	Donor number	Gender	Age	Diagnosis
Concomitant with reaming	1	Male	40	IM nailing of closed tibial shaft fracture
Concomitant with reaming	2	Male	28	IM nailing of closed tibial shaft fracture
Concomitant with reaming	3	Female	46	IM nailing of closed tibial shaft fracture
Concomitant with reaming	4	Male	30	IM nailing of closed tibial shaft fracture
Concomitant with reaming	5	Female	53	IM nailing of closed tibial shaft fracture
Concomitant with reaming	6	Male	18	IM nailing of closed tibial shaft fracture
Concomitant with reaming	7	Male	21	IM nailing of closed tibial shaft fracture
Concomitant with reaming	8	Female	53	IM nailing of closed tibial shaft fracture
Concomitant with reaming	9	Male	73	IM nailing of closed tibial shaft fracture
Post-reaming	1	Male	19	Nonunion femur
Post-reaming	2	Female	50	Nonunion femur
Post-reaming	3	Male	22	Nonunion femur
Post-reaming	4	Female	64	Nonunion tibia
Post-reaming	5	Male	33	Nonunion femur
Post-reaming	6	Male	43	Nonunion femur
Post-reaming	7	Male	62	Nonunion tibia
Post-reaming	8	Male	63	Nonunion femur
Post-reaming	9	Female	58	Nonunion tibia
Post-reaming	10	Female	44	Nonunion tibia
Post-reaming	11	Male	38	Nonunion tibia
Post-reaming	12	Female	24	Nonunion tibia
Post-reaming	13	Female	50	Non union tibia
Post-reaming	14	Female	45	Non union tibia
Post-reaming	15	Male	66	Non union radius
Prior surgery	1	Female	39	Sacroilitis for sacroiliac screws
Prior surgery	2	Male	67	Removal of metal work ankle
Prior surgery	3	Male	62	Removal of metal work tibia
Prior surgery	4	Female	42	Sacroilitis for sacroiliac screws
Prior surgery	5	Male	21	Removal of metal work tibia
Prior surgery	6	Male	18	Osteomyelitis tibia for debridement
Prior surgery	7	Female	46	Coccyxdinea for local injection
Prior surgery	8	Male	18	Osteomyelitis tibia for debridement
Prior surgery	9	Male	42	Sacroilitis for sacroiliac screws
Prior surgery	10	Male	41	Meniscal tear of knee for arthroscopy
Prior surgery	11	Female	31	Sacroilitis for sacroiliac screws
Prior surgery	12	Male	38	Osteomyelitis tibia for debridement

Supplementary Table 2. Rheumatoid arthritis patient information

RA category	Donor number	Sex	Age	CRP (mg/l)	ESR (mm/h)	Plasma viscosity (mPa.s)	RF (Iu/ml)	AntiCCP (U/ml)
Early	1	Female	77	<5.0	11	1.53	66	1.5
Early	2	Female	37	27	39	na	<15	0.4
Early	3	Female	41	<5.0	12	na	41	7.7
Early	4	Female	59	34	42	na	63	1.1
Early	5	Male	70	<5.0	10	na	210	101
Early	6	Male	74	28	na	1.73	65	>300.0
Early	7	Female	51	<5.0	na	1.74	<15	2.10
Early	8	Male	49	56	na	2.03	<15	1.70
Early	9	Female	73	54	9	na	22	16
Early	10	Male	73	<5.0	9	na	<15	2.6
Early	11	Female	49	<5.0	9	na	115	7.3
Early	12	Female	23	46	7	na	31	1.0
Early	13	Female	52	23	na	na	425	24.4
Early	14	Female	48	<5	na	na	624	77.30
Early	15	Female	51	17.9	56	1.81	71	>300.0
Established	1	Female	45	10.1	59	na	<15	>300.0
Established	2	Female	41	<5	9	1.54	15	0.50
Established	3	Male	56	<5	3	na	60	>300.00
Established	4	Female	47	<5	24	1.74	<15	<0.50
Established	5	Male	66	<5	8	na	119	>300.0
Established	*6	Female	34	5.9	26	na	51	1.6
Established	7	Female	42	<5	na	na	52	10.9
Established	8	Male	48	<5	na	na	234	>300.0
Established	9	Female	54	<5	50	na	98	253.5
Established	10	Male	54	<5	5	na	16	>300.0
Established	11	Female	32	<5	na	1.45	<15	111.2

Early RA is defined by < 1 year disease duration. CRP = C-reactive protein, ESR = erythrocyte sedimentation rate, RF = rheumatoid factor, CCP = cyclic citrullinated peptide. na = not available. * yielded 1 colony

Supplementary Table 3. Taqman assays used

Taqman assay identification	Gene Symbol	Description
Hs00909449_m1	***ACTA2***	actin, alpha 2, smooth muscle, aorta
Hs00244715_m1	***ACVR1B***	activin A receptor, type IB
Hs00155658_m1	***ACVR2A***	activin A receptor, type IIA
Hs00758162_m1	***ALPL***	alkaline phosphatase
Hs00181613_m1	***ANGPT1***	angiopoietin 1
Hs01101127_m1	***ANGPTL4***	angiopoietin-like 4
Hs03044164_m1	***BAMBI***	BMP and activin membrane-bound inhibitor homolog
Hs00609452_g1	***BGLAP***	bone gamma-carboxyglutamate (gla) protein/ osteocalcin
Hs00154192_m1	***BMP2***	bone morphogenetic protein 2
Hs00234930_m1	***BMP5***	bone morphogenetic protein 5
Hs00403062_m1	***BMPER***	BMP binding endothelial regulator
Hs00831730_s1	***BMPR1A***	bone morphogenetic protein receptor, type IA
Hs00156438_m1	***CDH11***	cadherin 11, type 2, OB-cadherin (osteoblast)
Hs00269972_s1	***CEBPA***	CCAAT/enhancer binding protein (C/EBP), alpha
Hs00166657_m1	***COL10A1***	collagen, type X, alpha 1
Hs01076777_m1	***COL1A1***	collagen, type I, alpha 1
Hs01028971_m1	***COL1A2***	collagen, type I, alpha 2
Hs00264051_m1	***COL2A1***	collagen, type II, alpha 1
Hs00426981_m1	***CSPG4***	chondroitin sulfate proteoglycan 4/ NG2
Hs00171022_m1	***CXCL12***	chemokine (C-X-C motif) ligand 12/ stromal cell-derived factor 1
Hs00322497_m1	***DAAM2***	dishevelled associated activator of morphogenesis 2
Hs00178815_m1	***DDR2***	discoidin domain receptor tyrosine kinase 2
Hs00182901_m1	***DVL2***	dishevelled, dsh homolog 2
Hs00358886_m1	***EFNA1***	ephrin-A1
Hs00174752_m1	***EPHB4***	EPH receptor B4
Hs00191912_m1	***EPYC***	epiphycan
Hs00609791_m1	***FABP4***	fatty acid binding protein 4, adipocyte
Hs00170454_m1	***FGF5***	fibroblast growth factor 5
Hs00241111_m1	***FGFR1***	fibroblast growth factor receptor 1
Hs00179829_m1	***FGFR3***	fibroblast growth factor receptor 3
Hs00173503_m1	***FRZB***	frizzled-related protein/ secreted frizzled-related protein 3
Hs00268943_s1	***FZD1***	frizzled homolog 1
Hs00201853_m1	***FZD4***	frizzled homolog 4
Hs00275833_s1	***FZD7***	frizzled homolog 7
Hs00259040_s1	***FZD8***	frizzled homolog 8
Hs00268954_s1	***FZD9***	frizzled homolog 9
Hs99999905_m1	***GAPDH***	glyceraldehyde-3-phosphate dehydrogenase
Hs00231119_m1	***GATA2***	GATA binding protein 2
Hs01075601_m1	***GHR***	growth hormone receptor
Hs00748445_s1	***GJA1***	gap junction protein, alpha 1, 43kDa/ Connexin 43
Hs99999909_m1	***HPRT1***	hypoxanthine phosphoribosyltransferase 1
Hs01005963_m1	***IGF2***	insulin-like growth factor 2 (somatomedin A),INS-IGF2
Hs00181211_m1	***IGFBP3***	insulin-like growth factor binding protein 3
Hs00961622_m1	***IL10***	interleukin 10
Hs00174202_m1	***IL7***	interleukin 7

Hs01070036_m1	***JAG1***	jagged 1
Hs00174492_m1	***LEPR***	leptin receptor
Hs01012571_m1	***LPL***	lipoprotein lipase
Hs00174838_m1	***MCAM***	melanoma cell adhesion molecule/ CD146
Hs00427183_m1	***MSX1***	msh homeobox 1
Hs02379661_g1	***MT2A***	metallothionein 2A
Hs00159522_m1	***MYH9***	myosin, heavy chain 9, non-muscle
Hs02387400_g1	***NANOG***	Nanog homeobox
Hs00707120_s1	***NES***	nestin
Hs00182120_m1	***NGFR***	nerve growth factor receptor (TNFR superfamily, member 16)/ CD271
Hs00918411_s1	***NGFRAP1***	nerve growth factor receptor (TNFRSF16) associated protein 1
Hs00271352_s1	***NOG***	noggin
Hs00192325_m1	***OMD***	osteomodulin
Hs00170179_m1	***PCOLCE***	procollagen C-endopeptidase enhancer
Hs00998018_m1	***PDGFRA***	platelet-derived growth factor receptor, alpha polypeptide
Hs00185122_m1	***PDGFRL***	platelet-derived growth factor receptor-like
Hs01370291_g1	***PHOSPHO1***	phosphatase, orphan 1
Hs00999632_g1	***POU5F1***	POU class 5 homeobox 1 (Oct 4)
Hs01115513_m1	***PPARG***	peroxisome proliferator-activated receptor gamma
Hs00231079_m1	***RUNX1***	runt-related transcription factor 1
Hs00231692_m1	***RUNX2***	runt-related transcription factor 2
Hs00173499_m1	***S1PR1***	sphingosine-1-phosphate receptor 1
Hs00610060_m1	***SFRP1***	secreted frizzled-related protein 1
Hs00180066_m1	***SFRP4***	secreted frizzled-related protein 4
Hs00361747_m1	***SORT1***	sortilin 1
Hs01053049_s1	***SOX2***	SRY (sex determining region Y)-box 2
Hs00165814_m1	***SOX9***	SRY (sex determining region Y)-box 9
Hs00277762_m1	***SPARC***	secreted protein, acidic, cysteine-rich/ osteonectin
Hs00959010_m1	***SPP1***	osteopontin/ secreted phosphoprotein 1/ bone sialoprotein I
Hs00559661_m1	***TGFBR2***	transforming growth factor, beta receptor II (70/80kDa)
Hs00234257_m1	***TGFBR3***	transforming growth factor, beta receptor III
Hs00900360_m1	***TNFRSF11B***	tumor necrosis factor receptor superfamily, member 11b/ osteoprotegerin
Hs00361186_m1	***TWIST1***	twist homolog 1
Hs00382379_m1	***TWIST2***	twist homolog 2
Hs00900058_m1	***VEGFA***	vascular endothelial growth factor A
Hs01099206_m1	***VEGFC***	vascular endothelial growth factor C
Hs00183662_m1	***WIF1***	WNT inhibitory factor 1

HPRT was used as the reference gene; *GAPDH* was used as a technical internal control only

Supplementary Figure 1A (*ACTA1-GATA2*) Relative gene expression in MSCs of different origin

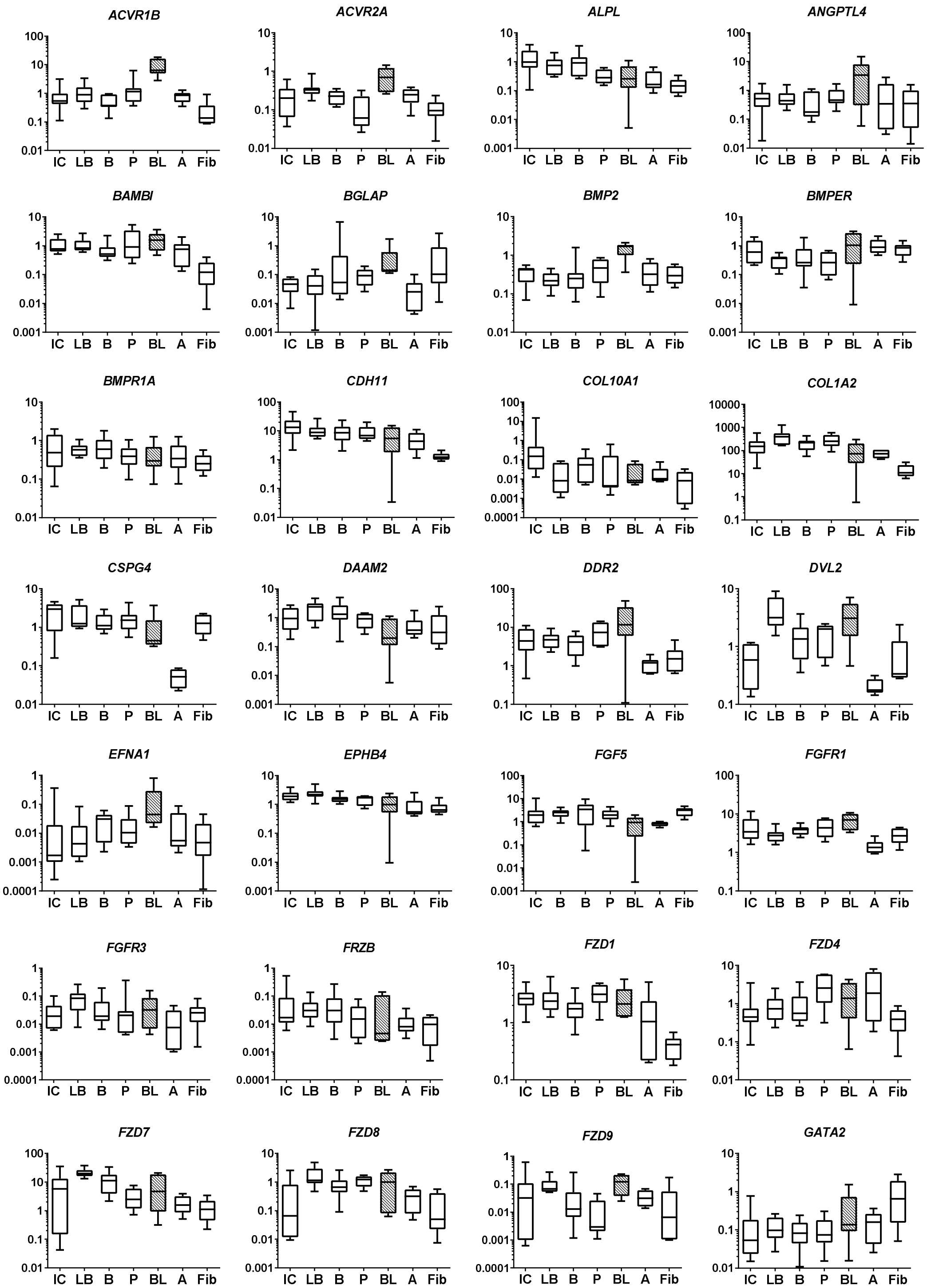

Gene expression normalised to *HPRT* using $2^{-\Delta Ct}$. IC=iliac crest marrow, LB=long-bone marrow, B=control bone, P=periosteum, BL=blood, A=adipose, Fib=fibroblast

Supplementary Figure 1B (*GHR-WIF1*) Relative gene expression in MSCs of different origin

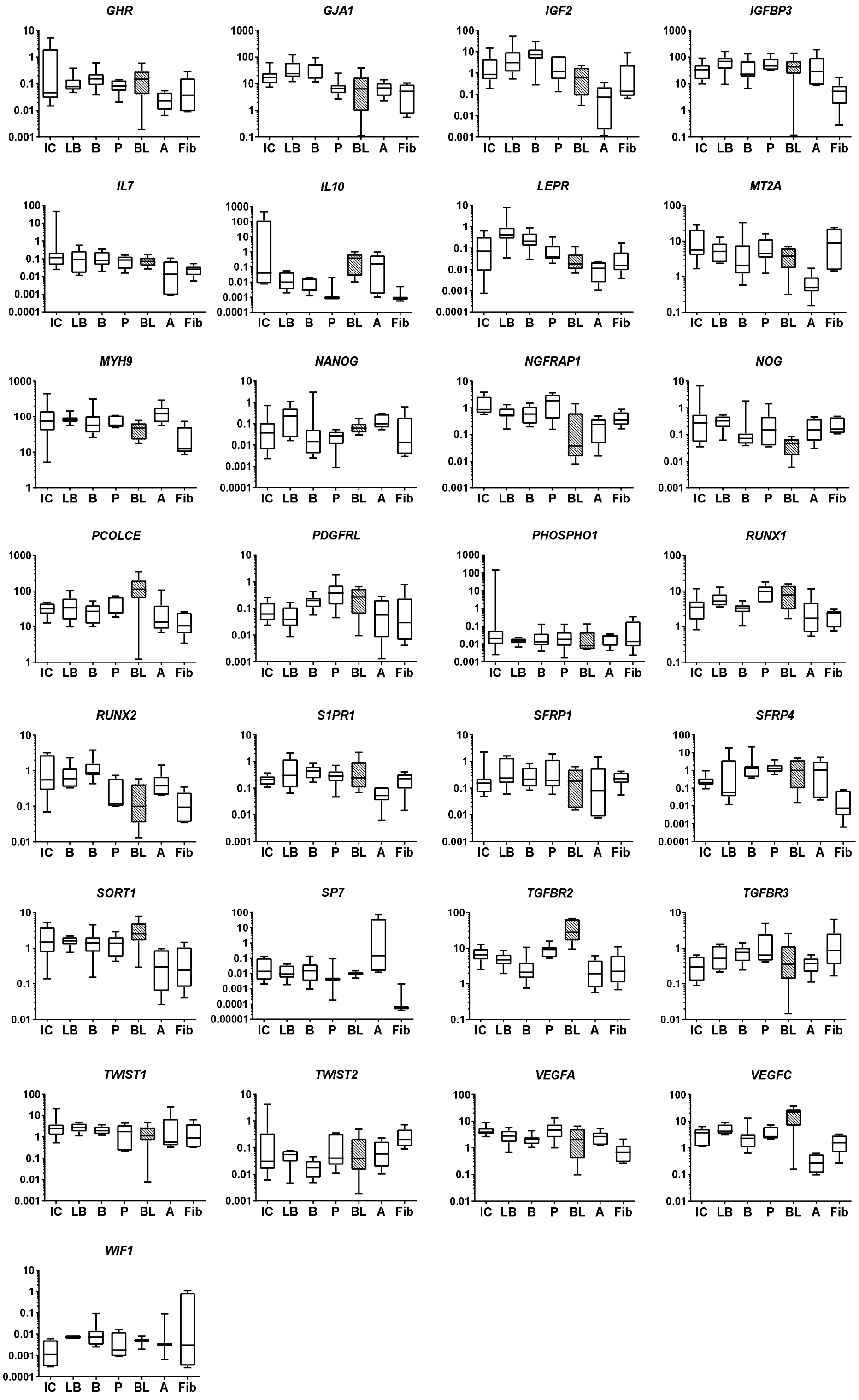

Gene expression normalised to *HPRT* using $2^{-\Delta Ct}$. IC=iliac crest marrow, LB=long-bone marrow, B=control bone, P=periosteum, BL=blood, A=adipose, Fib=fibroblast

Supplementary Figure 2 Thirty-one genes with no overlapping gene expression between PVB- and FVB-MSCs

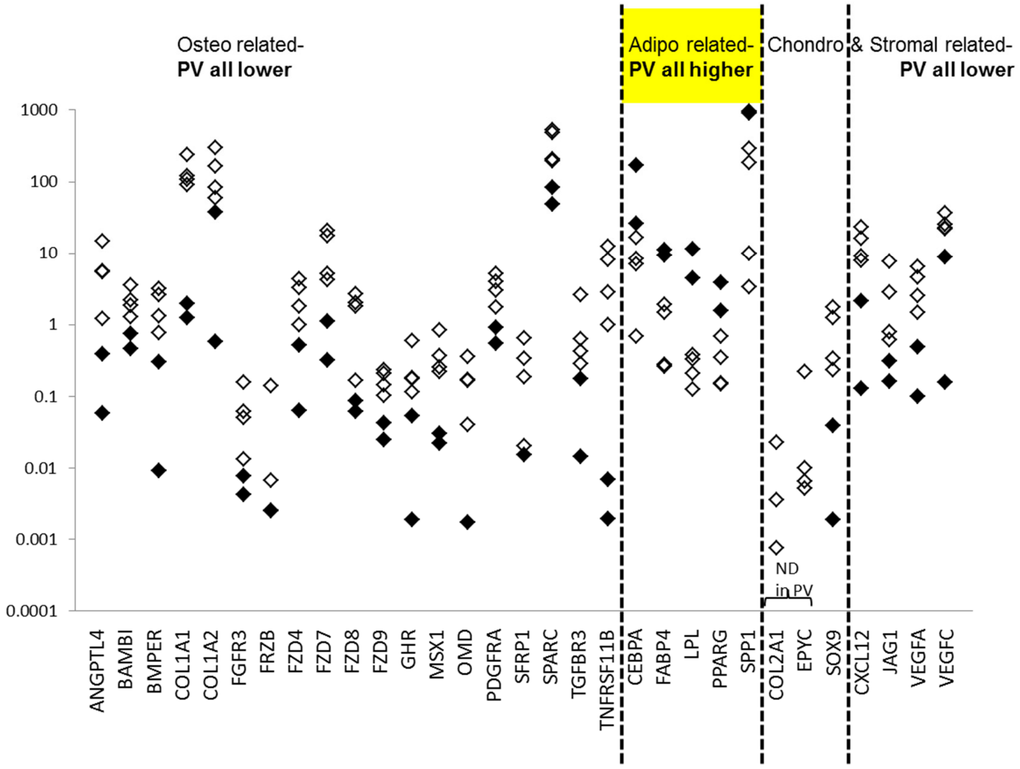

Black diamonds = PVB-MSCs, open diamonds FVB-MSCs. ND = not detected
